# Hysterical Aphonia

**Published:** 1877-06

**Authors:** G. Whitefield Ward

**Affiliations:** Late Clinical Assistant to the London Throat Hospital, etc., etc.; 123 West 11th St., N. Y. City


### HYSTERICAL APHONIA.

By G. Whitefield Ward, A.M., M.D.

[Late Clinical Assistant to the London Throat Hospital, etc., etc.]

There is no subject in the whole history of medicine so open to controversy as hysteria. One author defines it, "a perversion of the will;" another, "an inability or indisposition to exert the will;" whilst a third, denying that it is a special disease, says that it is "merely a symptom," and defines it as "a series of phenomena occurring in the course of a disease, sometimes known and sometimes obscure." Louyer-Villermy, decidedly holds that every hysterical woman is short, stout, dark, plethoric, and full of life and health. Sydenham, White, Copeland, and English writers in general, represent the hysterical predisposition with almost the very opposite characteristics. Whilst Briquet says the disease takes women as it finds them, blondes, brunettes, stout, thin, weak, ruddy, or pale, there is no choice. My idea in giving these different opinions is to show how impossible it would be for any one to form a correct view of hysteria by a perusal of the literature current upon the subject. Taking this fact into consideration, it is not at all surprising to me that writers have hitherto confounded hysterical loss of voice with the so-called functional aphonia, setting down hysteria as a prime cause of the latter, and I shall endeavor, in the present article, to rescue hysterical aphonia from the mire in which it has inadvertantly fallen, and place it upon a firm and substantial basis of its own.

Definition: *Hysterical aphonia* is that species of loss of voice which is due to some defect or perversion of will power presiding over the physiological action of the muscles of the vocal cords, which, for the time being, causes a false or simulated paralysis of these muscles.

*Functional aphonia* is that species of loss of voice, which is due to a true paralysis of one or more of the muscles controling the action of the cords, and preventing their approximation or their due tension when in apposition. As may readily be inferred from the first of the two definitions given above, the theory which I advance, and shall endeavor to prove, with regard to a case of pure, uncomplicated hysterical aphonia, is, that there is not the slightest paralysis of any of the muscles controling the action of the cords, that the muscles themselves, and the nerves presiding over their action are in a perfectly normal condition, and that the trouble lies with volition, which, from some cause or other, is deficient or perverted.

*Hysterical aphonia* is most liable to occur in the single female at or about the time of the commencement of puberty. The luxurious and lazy habits peculiar to a certain class among the wealthy, a class that have literally nothing to occupy their minds, except the perusal of silly novels, and the performance of the sillier things that "society" prescribes, conduce to the development of hysteria. The exciting cause is generally some sudden emotional disturbance; as shock, caused by sudden death of a near relative; fright brought about by some impending calamity, as shipwreck; or anxiety produced by the continued absence of a missing husband or child.

*Functional aphonia* is liable to occur in both sexes and among all classes of society, from the richest to the poorest, although one type of it, namely, that dependent upon anæmia, is more apt to overtake poorly-nourished working girls, especially that class who are obliged to sit through long and weary hours in closely-confined badly-ventilated apartments. A paresis of one or more muscles of the cords, from a prolonged bad usage of the voice, is common to all trades and professions, especially those that require a continuous loud talking, or, more properly speaking, a shouting in the open air, as the auctioneer, the stump speaker, the hawkster, &c. The same thing may happen to a singer from a sudden strain of the cords during too great an effort at vocalization.

*Symptomatology.* The will is perverted and defective, while ideas exhibit excessive activity. The hysterical patient says that she cannot speak aloud, and while under the belief that the action is impossible, it is so, although it may be obvious that the patient is pretending, or acting a part, for the time being; it is also often true that the hysterical patient states the fact. What she wants is motive, which may often be supplied by a sudden alarm or accidental circumstance. This symptom, or idea, is not confined to talking, but is manifested in other voluntary acts; one person says she cannot move this or the other limb, another that she cannot stand or walk, a third cannot open her eyes. But often, under the influence of some unexpected event, they do the very things that were said to be impossible. Hammond says that "hysteria is a partial insanity—an insanity, however, in which the patient does not entirely lose control, and which is capable of being overcome by the voluntary effort of the patient, provided a sufficient stimulus to normal condition be brought to bear. It thus happens that through the influence of such stimulus every symptom of hysteria disappears as if by magic." If you examine the larynx of a person suffering from pure, uncomplicated hysterical aphonia, you will see that the throat is healthy, or is simply relaxed, the vocal cords being widely separated, and slight effort being made for their approximation. The patient makes no attempt to whisper aloud; the failure is evidently not in the apparatus of the voice, as a mechanical production, but in the will to put that machinery into play. A common symptom is that of a ball, or lump, in the throat, a something which the patient cannot swallow, and which she feels will choke her. It seems to her as if something were tight there; she makes constant attempts to swallow, but the lump will not move. Mental and emotional conditions are apt to be exaggerated in a great degree. I have frequently seen the mere introduction of the mouth mirror of the laryngoscope bring on paroxysms of immoderate and uncontrollable laughter, which seem to have been excited by sensations of tickling experienced by the patient. I have also seen the same manipulation set up a prolonged fit of sobbing, the patient asserting that the introduction of the mirror caused great pain.

If you examine the larynges in cases of aphonia dependent upon true paralysis of the vocal muscles, the parts will present a picture differing widely from that depicted in the hysterical type. If the tensors are involved, an impaired vibration will be observed; if the arytenoideus is affected, the glottis will be widely open posteriorly; if the combined action of the crico-arytenoideus lateralis, and the arytenoideus is prevented, the vocal cords will remain widely separated, without any appearance of coming together. The general appearance and condition of the internal structures of the larynx in this latter class of cases will depend entirely upon the cause; if from anæmia, there will be a diffused paleness, not, however, confined to the windpipe, but extending upwards into the fauces and mouth, especially marked in the mucous membrane covering the hard palate; if from overstrain of the voice, whether from acts of singing or speaking, the vocal cords are apt to be found congested and mottled red, with possibly a little thickening, the ventricular bands to be more highly colored than natural, and slightly enlarged, and the neighboring parts inflamed.

*Diagnosis.* If the symptoms already described be borne in mind, and the history of each case be carefully considered, there is not much difficulty in diagnosis. The form of true paralysis of the cords, which is most likely to be confounded with the hysterical type, is that in which the cords remain widely separated during an attempt at phonation, the difficulty being in the crico-arytenoideus lateralis and arytenoideus, the adductors of the vocal cords. These two conditions can be readily diagnosed if the following test be employed. When the mouth-mirror is in situ, direct your patient to cough; if the case is one of true paralysis, the cords will not be visibly affected by the act of coughing; if it is one of hysterical or false paralysis, then the cords will be seen to adduct and approach each other in the median line. This forced adduction will invariably occur in connection with hysterical loss of voice. Another diagnostic point, and one that will instantly set all doubt aside, if it is present, is, that frequently, when undergoing a laryngoscopic examination, the hysterical patient will forget all about her infirmity, and utter, in a natural tone, the vowels a and e, when requested to do so by the examiner, the excitement of the moment being sufficient to divert the sufferer's attention from the throat, and allow volition to act normally. I call to mind an interesting case, illustrative of the above point, which occurred to me in my practice a little less than a year ago. The patient in question, a young lady æt. 18, suddenly became aphonic, about two weeks prior to my seeing her. The history which I elicited at the time established the fact that she was of an hysterical diathesis, but threw no light upon the exciting cause of the aphonia. She had always been of a nervous and excitable temperament, and was in the habit of losing control of her emotions at the most trivial circumstance. I further learned that the patient was subject to attacks of aphonia, having lost her voice several times before, but that the lost power had, in each instance, returned within a week.

*Laryngoscopic examination.* The larynx was found to be in a perfectly healthy condition, likewise the fauces and mouth. On requesting the patient to say a, to my great surprise, she did so, speaking in a clear and loud tone, although but a moment before she had postively asserted that she could not talk above a whisper. During the above act, both cords were distinctly seen to adduct and approach each other normally, in the middle of the air tube. Upon withdrawing the mouth mirror, and again requesting my patient to say a, she could not give utterance to that letter above a whisper, showing clearly that the cords were not being adducted. Another laryngoscopic examination was made with the same result as the first, the voice being restored, only to be lost again on the withdrawal of the mirror. Not fully appreciating the symptomatology of this case, I applied a mild astringent to the cords, and requested the patient to call again. On the occasion of her next visit, I followed exactly the same course in my examination as above described, with exactly the same results. Feeling confident that no ordinary plan of treatment would ever restore her voice, and that some sudden shock was necessary to produce the desired effect, I determined to invoke the aid of electricity; to this end I made use of the strongest current obtainable from a single-celled electro-magnetic apparatus, applying the negative pole externally over the thyroid cartilage, by means of an ordinary sponge electrode, and the positive pole directly upon the vocal cords, by means of Mackenzie's laryngeal electrode. Immediately, on receipt of the shock, the patient made violent efforts to escape, wildly gesticulating, and screaming outright, and I was compelled to withdraw the instrument, having retained it within the larynx for a few seconds only. This one somewhat severe application completely restored the voice of this patient, and the cure has been permanent up to the time I last met her—a fortnight ago. I confess that this case completely mystified me at the time, my ideas of hysteria, both as regards its pathology and histology, being very vague. It also stimulated me to a desire of more thoroughly understanding and comprehending the various phases developed in connection with hysteria, especially in its relations to aphonia. To this end, I made a careful examination, both subjectively and objectively, of all cases of aphonia which applied for treatment at the "London Throat Hospital," during my term of service, which were set down as hysterical. In the above case, the mere act of introducing the mirror caused the patient to forget all about her lost power, and to unconsciously say ā. The slight diversion brought about at the time of the examination was sufficient to turn the perverted will power into its proper channel, and allow the delicate laryngeal muscles to adduct the cords in a normal manner. If the aphonia had been due to a true paralysis of the adductor muscles, would it have been possible for it to have been dispelled by such a slight circumstance as making a laryngeal examination? By no means; the deficient muscular action would have to be restored by a regular and judicious course of treatment, applied with especial reference to the cause, and no diversion of mind or shock of any description would restore the lost power. When I withdrew the mirror and spoke to the patient, she answered me in a whisper and could not, or would not, speak aloud; there being nothing to occupy her mind, the one idea, the lost power, predominated.

Another very instructive and most remarkable case of hysterical aphonia came under my notice at the "London Throat Hospital."

The patient, a woman æt. 24, had suffered with complete loss of voice, of six months duration. On the day of her application for treatment, she started out with an associate to walk to the hospital, a distance of about two miles. During her journey thither, she quite alarmed her companion by addressing her in conversation, speaking aloud for the first time in six months. On questioning her at the hospital, prior to a laryngoscopic examination, she declared that her voice was as good as it had ever been. A careful examination elicited nothing except a little paleness of the ventricular bands and neighboring parts, and the cords were found to act perfectly well in every respect. Having applied a mild current of electricity direct to the cords, and ordered a mixture composed principally of ammoniae carbonas, the patient was dismissed, apparently cured, and as she did not return during my stay there, I conclude that her recovery was permanent. I do not think it could be possible to produce any combination of cases so remarkably illustrative of the affection to which I have devoted these pages as the two enumerated above. Hysterical manifestations developed in other parts of the body than the throat, seem to me to afford further proof of the correctness of the theory which I have advanced. J. Russell Reynolds, in "Reynolds' Practice," comparing hysterical paralysis of the lower extremities with true paralysis of these organs, says "that the ataxic or paraplegic patient tries to walk; the hysterical girl tries to show that she cannot use her limbs; if the former forgets himself, he falls; if the latter forgets herself, she walks." Most every experienced practitioner has met with cases of hysteria in his practice, in which the seemingly lost muscular power, whether of the arm, leg, or other member, could be completely restored, for the time being, by a little tact or ingenuity in absorbing the patient's attention, or directing her thoughts in a different channel. Hammond, in his "Diseases of the Nervous System," relates the case of a young lady who came under his charge for what was supposed to be a disease of the spinal cord; she had taken to her bed suddenly, soon after striking her back rather gently against the edge of the table, declaring she could not walk. On examination, he (Prof. Hammond) was convinced that there was no disease whatever of the spine other than that of a purely hysterical character, and so he expressed himself to her. She, nevertheless, insisted upon it that her spine was seriously injured, and she continued to keep her bed, lamenting daily her sad fate at being compelled to pass so long a time shut out from the enjoyments of life. There was no paralysis, for she moved her legs about freely enough in bed. But one evening, her brother, who had been long absent, returned home. She heard the bustle in the house attendant upon his arrival, but all were too busy to pay any attention to her in the chamber up stairs. Suddenly exclaiming, "I can stand this no longer," she sprang from her bed, rang for her maid, and hurrying on her clothes, proceeded down stairs, and entered the drawing-room, to the great surprise of all her family. The same author records another instructive case, namely, that of a lady who closed her eyes, and declared that she could not open them. She was brought to the professor as a case of double ptosis. He, after finding no evidence of such cerebral lesions as are generally met with in cases of ptosis, and having ascertained the fact that she was of an hysterical diathesis, expressed the opinion to her friends that the closure of her lids was not dependant on true paralysis of the muscles controling their action, but on hysteria, or, in other words, it was a hysterical simulation. The application of the induced current proved so disagreeable, not to say painful, to the patient, that two applications were sufficient to restore her volitionary power so that she opened her eyes without difficulty. Savory, in an article on hysteria, in "Holmes' Surgery," says "that hysteria frequently occurs strangely disguised, and its essential features concealed in the garb of some other disease whose characters it assumes and whose symptoms it mimics. This it is which specially gives interest and importance to hysteria, which so often renders it a stumbling-block in the way of a correct diagnosis, frequently the similarity of its symptoms leading to the error of mistaking a simulated for a real disease. And this form of hysteria has been aptly termed protean, for there are few diseases the symptoms of which are not at times more or less mimicked by this singular affection."

Therapeutically, the differentiation of hysterical aphonia from the functional variety is of vast importance, not only to the success of the practitioner, but also to the welfare of the patient, for if a case of ordinary functional aphonia be set down as hysterical, and the physician start with the idea that the paralysis is simulated, he is apt to pursue a too harsh plan of treatment, namely, the undue use of electricity, which will have a tendency rather to increase than to diminish the difficulty. In functional aphonia, the continued and systematic employment of mild currents of electricity, combined with astringent applications to the cords, and the internal administration of tonics, such as quinia and iron, if there be co-existent anæmia, will in nearly every instance, effect a cure. In hysterical aphonia, a sudden, powerful shock of electricity will generally restore the voice, and frequently a second application will not be necessary, the fear of a repeated dose proving sufficient stimulus to volition to allow it to act in a normal manner.

123 West 11th St., N. Y. City.

